# Altered Regional Homogeneity in Rolandic Epilepsy: A Resting-State fMRI Study

**DOI:** 10.1155/2014/960395

**Published:** 2014-08-28

**Authors:** Ye-Lei Tang, Gong-Jun Ji, Yang Yu, Jue Wang, Zhong-Jin Wang, Yu-Feng Zang, Wei Liao, Mei-Ping Ding

**Affiliations:** ^1^Department of Neurology, the Second Affiliated Hospital of Medial College, Zhejiang University, No. 88 Jiefang Road, Hangzhou 310009, China; ^2^Center for Cognition and Brain Disorders and the Affiliated Hospital, Hangzhou Normal University, Hangzhou 310015, China; ^3^Zhejiang Key Laboratory for Research in Assessment of Cognitive Impairments, Hangzhou 310015, China

## Abstract

Children with rolandic epilepsy (RE) are often associated with cognitive deficits and behavioral problems. Findings from neurophysiological and neuroimaging studies in RE have now demonstrated dysfunction not only in rolandic focus, but also in distant neuronal circuits. Little is known, however, about whether there is distributed abnormal spontaneous brain activity in RE. Using resting-state functional magnetic resonance imaging (RS-fMRI), the present study aimed to determine whether children with RE show abnormal local synchronization during resting state and, if so, whether these changes could be associated with the behavioral/clinical characteristics of RE. Regional homogeneity (ReHo) in children with RE (*n* = 30) and healthy children (*n* = 20) was computed on resting-state functional MRI data. In comparison with healthy children, children with RE showed increased ReHo in the central, premotor, and prefrontal regions, while they showed decreased ReHo in bilateral orbitofrontal cortex and temporal pole. In addition, the ReHo value in the left orbitofrontal cortex negatively was corrected with performance intelligence quotient in the children with RE. The aberrant local synchronization, not strictly related to primary site of the typical rolandic focus, indicates the neuropathophysiological mechanism of RE. The study findings may shed new light on the understanding of neural correlation of neuropsychological deficiencies in the children with RE.

## 1. Introduction

Rolandic epilepsy (RE) is an idiopathic focal epilepsy syndrome, which occurs in childhood [1], and results in clinical manifestations of biphasic sharp wave discharges around the rolandic fissure [2, 3]. Since nearly 90% of these children with or without an antiepileptic drug spontaneously remits from seizures before puberty [4], RE is also known as a benign childhood epilepsy. However, children with RE are usually associated with a variety of cognitive disturbances [5, 6], and the underlying pathophysiological mechanisms remain largely unknown.

Although it is a focal epilepsy, findings from neuroimaging studies demonstrate dysfunction not only in rolandic focus but also in distant neuronal circuits. Recent studies using quantitative structural magnetic resonance imaging have shown widespread morphological changes in RE [7–11]. In addition, diffusion tensor imaging examinations have revealed alterations of white matter tracts' integrity [12–15]. Taken together, these structural aberrances associated with their cognitive abnormalities may reflect the progress of long-term impairment. Functional magnetic resonance imaging (fMRI) studies with simultaneously recorded electroencephalogram (EEG), on the other hand, have found concordant focal spike-associated blood-oxygen-level-dependent (BOLD) activation in perisylvian, central, premotor, and prefrontal regions, and all these findings are well corresponding to a typical seizure semiology [16–20]. However, a few studies found the findings of spike-related BOLD deactivation [21, 22], while others did not.

Recently, resting-state fMRI (rs-fMRI) techniques have been applied to demonstrate intrinsic abnormalities in various types of epilepsy [23–31]. Regional homogeneity (ReHo), as one of the rs-fMRI methods, could measure the functional coherence or synchronization of a given voxel with its nearest voxels, reflecting the local synchronization of the spontaneous BOLD fluctuations [32]. This local synchronization has neurobiological relevance that is likely determined by anatomical, developmental, and neurocognitive factors [33]. Thus, ReHo would serve as a neuroimaging marker to investigate the intact and/or abnormal brain function [34]. It may be speculated that an abnormal ReHo may be a clue to disrupted local functionality and may provide insight into the pathophysiology of brain disorder [35]. Thus, this method has been suggested to investigate the functional modulations and to characterize the neuropsychological changes in the resting state in patients with various clinical populations [36–42]. In particular, abnormal ReHo has mostly been used to depict aberrant spontaneous brain temporal synchrony in epilepsy [43–47]. Little is known, however, about the changes of local synchronization of spontaneous BOLD fluctuations in RE.

Based on previous EEG-fMRI findings regarding spike-related brain functional alterations in RE, we expect to find disrupted local synchronization of spontaneous BOLD fluctuations. We hypothesized that abnormal local synchronization persisted in RE during the interictal period and might be associated with neuropsychological deficiencies. Thus, the aim of the current study was to determine whether children with RE show abnormal local synchronization during resting state and, if so, whether these changes were correlated with the behavioral/clinical characteristics of RE. The purpose of this work was to delineate the neurophysiologically significant abnormal synchronous neuronal activity and neural correlation with neuropsychological deficiencies in the children with RE.

## 2. Materials and Methods

### 2.1. Participants

Thirty children whose conditions were diagnosed as RE (18 girls and 12 boys; all right-handed;, age [mean ± SD]: 9.60 ± 2.11 years) at the Second Affiliated Hospital of Zhejiang University School of Medicine were included prospectively in this study. Written informed consent was obtained from all parents. The study protocol was reviewed and approved by the Local Medical Ethics Committee of Center for Cognition and Brain Disorders, Hangzhou Normal University. The inclusion criteria for patients were as follows: (i) clinical and EEG findings evident of RE; (ii) aged between 6 and 13 years; (iii) attending regular schools; (iv) without developmental disabilities; (v) full-scale intelligence quotient (IQ) more than 70; and (vi) without history of addictions or neurologic diseases other than epilepsy. The patients' conditions were diagnosed on the basis of all available clinical and EEG data with the following criteria: (i) recommendations set by the International League against Epilepsy classification [48] and recent literature [2]; (ii) having simple partial, often facial, motor, or tonic-clonic seizures during sleep; and (iii) having spike-wave in centrotemporal regions, especially nocturnal interictal epileptiform discharges (IEDs) on EEG. Exclusion criteria were (i) focal abnormality in routine structural MRI examinations, (ii) falling asleep during rs-fMRI, and (iii) head motion parameters exceeding 3 mm in translation or 3 degrees in rotation.

Twenty sex- and age-matched healthy children controls (10 girls and 10 boys; all right-handed; age [mean ± SD]: 9.55 ± 2.14 years) were also included in the study. They had no history of neurologic disorders or psychiatric illnesses and no gross abnormalities on brain MR examinations. No significant difference in age (*T* = 0.08, *P* = 0.94) or gender (*χ* ^2^ = 0.70, *P* = 0.49) was found between groups. Demographic and clinical information is detailed in Table 1.

### 2.2. Simultaneous EEG-fMRI Acquisition

All patients underwent one or two simultaneous EEG-fMRI sessions to archive more IED as far as possible. Simultaneous EEG was not recorded in healthy controls. During fMRI acquisition, EEG data was continuously recorded with an MR-compatible EEG recording system (Brain Products, Germany). The 32 Ag/AgCl electrodes (through a 10/20 system) were attached to the scalp with conductive cream. Three electrooculogram/electrocardiogram channels were simultaneously recorded. Twenty-nine EEG electrodes were connected to a BrainAmp amplifier, with a sampling rate of 5 kHz. The amplifier was connected to the recording computer outside the scanner room through a fiber optic cable.

The EEG data was processed offline to filter out MR artifacts and to remove ballistocardiogram artifacts (Brain Vision Analyzer 2.0, Germany). IEDs were marked independently by two experienced electroencephalographers, according to both spatial distribution and morphology. Disagreements about the markers were resolved and consensuses were reached after discussion.

### 2.3. Neuropsychological Assessment

To test cognitive performance, a neuropsychological evaluation was administered. General intelligence was assessed using the Chinese version of Wechsler Intelligence Scale for Children (WISC-III), which included verbal IQ, performance IQ, and full-scale IQ. In addition, three factorial subscales of WISC-III were used to assess language comprehension, perceptual organization, and memory/attention. All scores were standardized for age and gender.

### 2.4. fMRI Data Acquisition

Functional and structural imaging data were acquired on a 3.0-Tesla MRI scanner (GE Discovery 750 MRI, General Electric, Milwaukee, WI, USA) at the Center for Cognition and Brain Disorders, Hangzhou Normal University. Foam padding was used to minimize head motion for all subjects. Functional images were acquired using an echoplanar imaging sequence (repetition time = 2000 ms, echo time = 30 ms, and flip angle = 90°). Thirty transverse slices (field of view = 220 × 220 mm^2^, in-plane matrix = 64 × 64, slice thickness = 3.2 mm, no interslice gap, and voxel size = 3.44 × 3.44 × 3.2 mm^3^) aligned along the anterior commissure-posterior commissure line were acquired. In each session, a total of 240 volumes were collected, resulting in a total scan time of 480 s. For each patient, one or two sessions were acquired. Subjects were instructed simply to rest with their eyes closed, not to think of anything in particular, and not to fall asleep. Subsequently, 3D T1-weighted anatomical images were acquired in the sagittal orientation using a magnetization prepared rapid acquisition gradient-echo sequence (repetition time = 8.06 ms, echo time = 3.136 ms, flip angle = 8°, field of view = 256 × 256 mm^2^, matrix size = 256 × 256, slice thickness = 1 mm, no interslice gap, voxel size = 1 × 1 × 1 mm^3^, and 176 slices) on each subject.

### 2.5. fMRI Data Preprocessing

Considering that the healthy controls underwent one session, the first session of RE was selected for further comparison. Preprocessing of functional images was carried out using DPARSF (http://www.restfmri.net) [49] and SPM8 (http://www.fil.ion.ucl.ac.uk/spm) toolkits. Functional images, after exclusion of the first 10 images, were initially corrected by slice-timing and realignment. No translation or rotation parameters in any given data set exceeded ±3 mm or ±3°. Moreover, the mean framewise displacement (FD) was computed by averaging FDi from every time point for each subject [50]. There were no differences for the mean FD between groups (*P* = 0.21) (Table 1). Individual 3D T1-weighted anatomical image was coregistered to functional images. The 3D T1-weighted anatomical images were segmented (grey matter, white matter, and cerebrospinal fluid). A nonlinear spatial deformation was then calculated from the grey matter images to a grey matter template in Montreal Neurological Institute space using 12 parameters affine linear transformation. This transformation was then applied to the functional images, which were resliced at a resolution of 3 × 3 × 3 mm^3^. Several sources of spurious variances (six head motion parameters, mean FD, global brain signal, and averaged signal from white matter signal and cerebrospinal fluid) were regressed out using a multiple linear regression analysis. Finally, data with linear trend were removed, and temporal band-pass was filtered (0.01–0.08 Hz).

### 2.6. ReHo Analysis

The similarity of the time series within a cluster was measured based on the regional homogeneity method [32]. The ReHo of the voxel at the center of the 27 nearest neighboring voxels cluster was calculated by Kendall's coefficient of concordance algorithm by REST software (http://www.restfmri.net) [51]. For standardization purposes, the individual ReHo map was divided by its whole brain mean ReHo value. Finally, the standardized ReHo maps were spatially smoothed with 4 mm of full width at half maximum isotropic Gaussian kernel.

### 2.7. Statistical Analysis

Differences in demographic and clinical data between RE children and healthy children were analyzed using a two-sample* t*-test and *χ* ^2^-test.

To investigate the differences in local synchronization between two groups, a two-sample* t*-test was performed on the individual standardized ReHo maps. Significant threshold was set at a corrected *P* < 0.05 (combined height threshold *P* < 0.01 and a minimum cluster size of 20 voxels) using the AlphaSim program in the REST software, which applied Monte Carlo simulation to calculate the probability of false positive detection by taking into consideration both the individual voxel probability thresholding and cluster size.

To explore the relationship between local synchronization and clinical behavior in children with RE, the averaged ReHo value of each sphere region of interests (centered at the peak voxel of each abnormal area, radius = 3 mm) was correlated with the clinical factor (epilepsy duration) and neuropsychological variables (including full-scale IQ, verbal IQ, and performance IQ) using Pearson correlation analysis on the patients group. The statistical threshold was set at *P* < 0.05.

## 3. Results

### 3.1. Neuropsychological Results

Demographic characteristics and neuropsychological scores are shown in Table 1. Children with RE had a significantly lower score of verbal IQ (*T* = 3.179, *P* = 0.003). There was no significant difference in full-scale IQ and performance IQ between the two groups (Table 1). There was also no significant correlation between IQ (full-scale IQ, verbal IQ, and performance IQ) and clinical characteristics (age of onset and duration of disease).

### 3.2. Between-Group ReHo Differences

**Figure 1:**
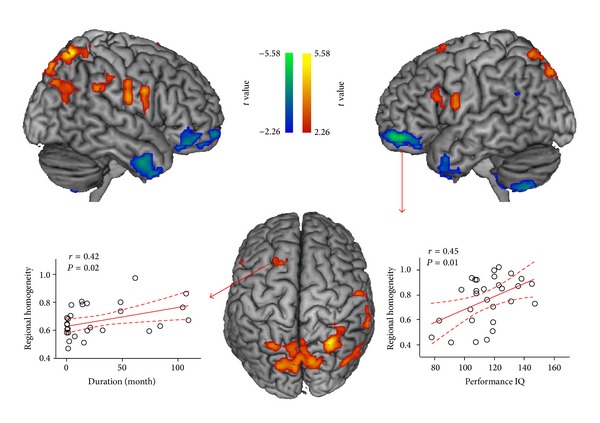
Brain regions showing abnormal regional homogeneity in children with rolandic epilepsy. Two-line graph indicated the local synchronization in left superior gyrus and left orbitofrontal area showing significant correlation with disease duration and performance IQ, respectively. The warm and cool colors indicate brain regions with increased and decreased regional homogeneity in children with RE, respectively.

The results obtained from the two-sample* t*-test showed significant differences in ReHo between two groups (*P* < 0.05, AlphaSim corrected; Figure 1, Table 2). Compared with healthy children, children with RE showed significantly increased ReHo in the bilateral precentral gyrus, right postcentral gyrus, right supramarginal gyrus, left inferior and superior frontal gyrus, and superior parietal lobule, while decreased ReHo was observed mainly in the bilateral temporal pole, bilateral orbitofrontal area, and putamen. 

### 3.3. Correlation between ReHo of Affected Areas with Clinical Features

Significant positive correlations were observed between the epilepsy duration and local synchronization in the left superior frontal gyrus (*r* = 0.42, *P* = 0.020). The performance IQ was negatively corrected with local synchronization in the left orbitofrontal area (*r* = 0.4569, *P* = 0.010) (Figure 1). Note that these two ROI's correlations have not survived multiple comparisons. There were no significant correlations between ReHo in the other abnormal areas and clinical and/or neuropsychological variables.

## 4. Discussion

To the best of our knowledge, this is the first study to examine local BOLD coherence in children with RE during resting state. Compared with healthy children, children with RE showed increased ReHo at the lower part of sensorimotor cortex and cortices around rolandic fissure, and they showed decreased ReHo in the limbic system. In addition, aberrant ReHo of several brain regions was associated with clinical or neuropsychological variables. The current findings extend understanding to the neuropathophysiological mechanisms of RE.

Epilepsy documents the altered neural substrates with hyperexcitable seizure networks [52]. Electrophysiological findings from both animal models and human brains have suggested an increased synchronization in the epileptogenic zone during ictal and interictal states [53, 54]. Therefore, it is worthwhile to investigate local synchronization of spontaneous fMRI BOLD signals in children with RE. Recently, ReHo was developed to characterize the coherence of spontaneous neuronal activity and was utilized to detect spontaneous brain dysfunction in various epileptic brains [43, 45, 46]. This study, using this method, aimed to test a hypothesis that the abnormal regional synchronization persists in children with RE in the interictal period.

Previous simultaneous EEG and fMRI studies found that interictal discharge could result in facial sensorimotor area involvement in RE seizures [17–22]. As expected, it was found that children with RE showed increased ReHo in the lower part of sensorimotor area and cortices around rolandic fissure. This is in line with the typical seizure semiology of RE that manifests paresthesia and jerking of the mouth, face, and hand [55]. Moreover, the study finding suggests that the abnormal function not only occurs during interictal discharges, but also exists throughout the interical period.

In children with RE, increased local BOLD synchronization was also observed in the left premotor cortex (Brodmann area 6) (Figure 1 and Table 2). The premotor cortex not only was involved in the planning of complex and coordinated movements, but also was associated with spatial attention and executive control [56]. Functionally, premotor cortex was connected with attention- and control-related networks in healthy juveniles and young adults. A previous study found that IEDs could cause hemodynamic changes in premotor regions in children with RE, indicating the motor and cognitive dysfunctions [21]. In the current study, deficits of BOLD coherence along with a positive correlation with disease severity provide evidence for a neural correlation of RE. In addition to the motor and premotor area, the cerebellum and striatum also play important roles in motor control. The decreased ReHo of these regions revealed their dysfunction in RE patients. It also suggested that the abnormal cortico-striato-cerebellar circuit might be related to the clinical syndrome of these patients.

We also observed increased ReHo in superior parietal lobule (SPL), which related to the posterior part of attention system [57]. Since SPL is posterior to the central area, we speculated that the increase might be a result of the propagation of the epileptic discharges [58]. Behavior study has revealed the impairment of spatial attention in RE patients [59]. Our findings complementally implicated the brain functional mechanism of this cognitive impairment. Future studies correlating with neurophysiology measurements with ReHo, the local neural synchronization, are warranted to validate such behavior-neuroimaging association.

The most remarkable finding in this study was the significant decrease in local synchronization in the limbic system (including bilateral orbitofrontal area and temporal pole) in the children with RE (Figure 1 and Table 2). A previous structural MRI study found subtle cortical abnormality in orbitofrontal region, suggesting the pathomorphology in the active phase of brain development in patients with RE [9]. However, this EEG-fMRI study did not find IED-related BOLD hemodynamic changes in this brain region, which could be attributed to a possibility that this might be a form of RE epilepsy with signs of cortical hyperexcitability that vary with time in terms of rate and side [19, 20]. The study results first provide the evidence of BOLD synchronization that the children with RE have disturbed orbitofrontal area functions. The human orbitofrontal cortex receives information from motor, limbic, and sensory cortices, reflecting sensory integration for executive motor control [60]. It could be speculated that sensory integration dysfunction in RE might be attributed to any harmful causes, for example, ictal epileptic activity, in addition to the neuronal inhibition induced by the IED activity. Moreover, this dysfunction was associated with performance IQ (Figure 1), which could imply an underlying neural correlation of neuropsychological deficiencies in children with RE.

The present work involved several limitations. First, the antiepileptic medication taken by some patients might confound the results; in future studies, homogeneous patients should be grouped more appropriately and the medication dose should be detailed. Second, the sample size used was modest; larger sample size may provide further insights. Third, we observed the IDE in only several patients. In this case, we did not analyze the IED-related BOLD hemodynamic changes in this group. Finally, the local synchronization was measured at a low sampling rate, which impeded investigating high rhythm alternation in RE. Future study should use advanced data acquisition sequences to enable whole brain fMRI scanning at subsecond temporal resolution [61].

## 5. Conclusions

The present study examined the local synchronization of BOLD fluctuation, providing a description of the pathology mechanism of RE. Children with RE showed increased regional homogeneity in central, premotor, and prefrontal regions, and the findings were consistent with the location of typical epileptic focus of RE. Children with RE also showed decreased regional homogeneity in the limbic system, not strictly related to primary site of the typical focus, suggesting impaired sensory integration in RE. The present results may shed new light on the understanding of neural correlation of neuropsychological deficiencies in children with RE.

## Figures and Tables

**Table 1:** Demographic and clinical characteristics of participants.

Characteristic	Patients (*n* = 30)	Controls (*n* = 20)	*P* value
Age (years)	9.60 ± 2.11	9.55 ± 2.14	0.935^a^
Sex (female/male)	18/12	10/10	0.485^b^
IQ			
Full-scale IQ	110.0 ± 14.95	116.2 ± 16.51	0.210^a^
Verbal IQ	103.5 ± 14.75	118.9 ± 16.94	**0.003** ^ a^
Performance IQ	115.2 ± 16.45	110.8 ± 15.75	0.378^a^
Education (years)	3.4 ± 1.96	4.2 ± 2.22	0.186^a^
Onset age (years)	7.53 ± 2.11	N.A.	—
Duration (months)	26.43 ± 35.66	N.A.	—
FD (mm)	0.18 ± 0.11	0.14 ± 0.08	0.210^c^

The intelligence quotient (IQ) scores in patients and controls were based on the results of 29 and 16 participants, respectively. FD denotes mean framewise displacement. The other values are illustrated as mean ± SD.

^a^Two-sample *t*-test.

^b^Chi-square test.

^c^Mann Whitney *U*-test.

**Table 2:** Brain regions showing abnormal regional homogeneity in patients with rolandic epilepsy.

Brain region	MNI coordinates (*XYZ*)	BA	*t* value	Voxel number
Patients > controls				
Precentral gyrus R.	48 3 30	4	4.73	44
Precentral gyrus L.	−51 3 24	4	3.96	31
Postcentral gyrus R.	58 −12 24	4/3	4.56	97
Inferior frontal gyrus L.	−51 18 24	45/46	3.35	31
Superior frontal gyrus L.	−18 12 72	6	3.70	22
Superior parietal lobule L.	12 −78 51	7	4.41	316
Superior parietal lobule R.	30 −63 63	7	5.59	87
Supramarginal gyrus R.	69 −39 33	40	4.47	44
Angular gyrus R.	45 −78 36	39	3.91	93
Patients < controls				
Temporal pole L.	−51 12 −33	38	−3.76	64
Temporal pole R.	54 0 −33	38/21	−4.10	162
Obitofrontal area L.	30 45 −12	11	−4.28	308
Obitofrontal area R.	−15 33 −18	11	−4.96	340
Angular gyrus L.	−42 −51 24	39	−4.26	20
Cerebellum	−12 −69 −33	—	−3.82	39
Cerebellum	−15 −63 −51	—	−5.17	430
Putamen R.	30 6 −6	—	−3.69	44

MNI: Montreal Neurological Institute; BA: Brodmann area; L: left; R: right.

## References

[1] Wirrell EC (1998). Benign epilepsy of childhood with centrotemporal spikes. *Epilepsia*.

[2] Panayiotopoulos CP, Michael M, Sanders S, Valeta T, Koutroumanidis M (2008). Benign childhood focal epilepsies: assessment of established and newly recognized syndromes. *Brain*.

[3] Guerrini R, Pellacani S (2012). Benign childhood focal epilepsies. *Epilepsia*.

[4] Stephani U, Carlsson G (2006). The spectrum from BCECTS to LKS: the rolandic EEG trait—impact on cognition. *Epilepsia*.

[5] Chan S, Lee W (2011). Benign epilepsy in children. *Journal of the Formosan Medical Association*.

[6] Camfield CS, Camfield PR (2014). Rolandic epilepsy has little effect on adult life 30 years later: a population-based study. *Neurology*.

[7] Sarkis R, Wyllie E, Burgess RC, Loddenkemper T (2010). Neuroimaging findings in children with benign focal epileptiform discharges. *Epilepsy Research*.

[8] Kanemura H, Hata S, Aoyagi K, Sugita K, Aihara M (2011). Serial changes of prefrontal lobe growth in the patients with benign childhood epilepsy with centrotemporal spikes presenting with cognitive impairments/behavioral problems. *Brain and Development*.

[9] Overvliet GM, Besseling RMH, Jansen JFA (2013). Early onset of cortical thinning in children with rolandic epilepsy. *NeuroImage: Clinical*.

[10] Lin JJ, Riley JD, Hsu DA (2012). Striatal hypertrophy and its cognitive effects in new-onset benign epilepsy with centrotemporal spikes. *Epilepsia*.

[11] Pardoe HR, Berg AT, Archer JS, Fulbright RK, Jackson GD (2013). A neurodevelopmental basis for BECTS: evidence from structural MRI. *Epilepsy Research*.

[12] Ciumas C, Saignavongs M, Ilski F (2014). White matter development in children with benign childhood epilepsy with centro-temporal spikes. *Brain*.

[13] Kim SE, Lee JH, Chung HK, Lim SM, Lee HW (2014). Alterations in white matter microstructures and cognitive dysfunctions in benign childhood epilepsy with centrotemporal spikes. *European Journal of Neurology*.

[14] Besseling RM, Jansen JF, Overvliet GM (2013). Reduced structural connectivity between sensorimotor and language areas in rolandic epilepsy. *PLoS ONE*.

[15] Xiao F, Chen Q, Yu X (2014). Hemispheric lateralization of microstructural white matter abnormalities in children with active benign childhood epilepsy with centrotemporal spikes (BECTS): a preliminary DTI study. *Journal of the Neurological Sciences*.

[16] Siniatchkin M, Moeller F, Jacobs J (2007). Spatial filters and automated spike detection based on brain topographies improve sensitivity of EEG-fMRI studies in focal epilepsy. *NeuroImage*.

[17] Boor R, Jacobs J, Hinzmann A (2007). Combined spike-related functional MRI and multiple source analysis in the non-invasive spike localization of benign rolandic epilepsy. *Clinical Neurophysiology*.

[18] Boor S, Vucurevic G, Pfleiderer C, Stoeter P, Kutschke G, Boor R (2003). EEG-related functional MRI in benign childhood epilepsy with centrotemporal spikes. *Epilepsia*.

[19] Masterton RAJ, Harvey AS, Archer JS (2010). Focal epileptiform spikes do not show a canonical BOLD response in patients with benign rolandic epilepsy (BECTS). *NeuroImage*.

[20] Masterton RAJ, Jackson GD, Abbott DF (2013). Mapping brain activity using event-related independent components analysis (eICA): specific advantages for EEG-fMRI. *NeuroImage*.

[21] Lengler U, Kafadar I, Neubauer BA, Krakow K (2007). fMRI correlates of interictal epileptic activity in patients with idiopathic benign focal epilepsy of childhood: a simultaneous EEG-functional MRI study. *Epilepsy Research*.

[22] Archer JS, Briellman RS, Abbott DF, Syngeniotis A, Wellard RM, Jackson GD (2003). Benign epilepsy with centro-temporal spikes: Spike triggered fMRI shows somato-sensory cortex activity. *Epilepsia*.

[23] Liao W, Zhang Z, Mantini D (2013). Dynamical intrinsic functional architecture of the brain during absence seizures. *Brain Structure and Function*.

[24] Liao W, Zhang Z, Mantini D (2013). Relationship between large-scale functional and structural covariance networks in idiopathic generalized epilepsy. *Brain Connect*.

[25] Liao W, Zhang Z, Pan Z (2010). Altered functional connectivity and small-world in mesial temporal lobe epilepsy. *PLoS ONE*.

[26] Liao W, Zhang Z, Pan Z (2011). Default mode network abnormalities in mesial temporal lobe epilepsy: a study combining fMRI and DTI. *Human Brain Mapping*.

[27] Zhang Z, Liao W, Wang Z (2014). Epileptic discharges specifically affect intrinsic connectivity networks during absence seizures. *Journal of the Neurological Sciences*.

[28] Zhang Z, Lu G, Zhong Y (2010). fMRI study of mesial temporal lobe epilepsy using amplitude of low-frequency fluctuation analysis. *Human Brain Mapping*.

[29] Zhang Z, Lu G, Zhong Y (2009). Impaired perceptual networks in temporal lobe epilepsy revealed by resting fMRI. *Journal of Neurology*.

[30] Zhang Z, Lu G, Zhong Y (2010). Altered spontaneous neuronal activity of the default-mode network in mesial temporal lobe epilepsy. *Brain Research*.

[31] Zhang Z, Lu G, Zhong Y (2009). Impaired attention network in temporal lobe epilepsy: a resting FMRI study. *Neuroscience Letters*.

[32] Zang Y, Jiang T, Lu Y, He Y, Tian L (2004). Regional homogeneity approach to fMRI data analysis. *NeuroImage*.

[33] Jiang L, Xu T, He Y (2014). Toward neurobiological characterization of functional homogeneity in the human cortex: regional variation, morphological association and functional covariance network organization. *Brain Structure and Function*.

[34] Zuo XN, Xu T, Jiang L (2013). Toward reliable characterization of functional homogeneity in the human brain: preprocessing, scan duration, imaging resolution and computational space. *NeuroImage*.

[35] He Y, Wang L, Zang Y (2007). Regional coherence changes in the early stages of Alzheimer's disease: a combined structural and resting-state functional MRI study. *NeuroImage*.

[36] Zhu C, Zang Y, Cao Q (2008). Fisher discriminative analysis of resting-state brain function for attention-deficit/hyperactivity disorder. *NeuroImage*.

[37] Liu Y, Wang K, YU C (2008). Regional homogeneity, functional connectivity and imaging markers of Alzheimer's disease: a review of resting-state fMRI studies. *Neuropsychologia*.

[38] Liu Z, Xu C, Xu Y (2010). Decreased regional homogeneity in insula and cerebellum: A resting-state fMRI study in patients with major depression and subjects at high risk for major depression. *Psychiatry Research—Neuroimaging*.

[39] Wu QZ, Li DM, Kuang WH (2011). Abnormal regional spontaneous neural activity in treatment-refractory depression revealed by resting-state fMRI. *Human Brain Mapping*.

[40] Wu T, Long X, Zang Y (2009). Regional homogeneity changes in patients with parkinson's disease. *Human Brain Mapping*.

[41] Paakki J, Rahko J, Long X (2010). Alterations in regional homogeneity of resting-state brain activity in autism spectrum disorders. *Brain Research*.

[42] Shukla DK, Keehn B, Müller RA (2010). Regional homogeneity of fMRI time series in autism spectrum disorders. *Neuroscience Letters*.

[43] Yang T, Fang Z, Ren J (2014). Altered spontaneous activity in treatment-naive childhood absence epilepsy revealed by regional homogeneity. *Journal of the Neurological Sciences*.

[44] Weaver KE, Chaovalitwongse WA, Novotny EJ, Poliakov A, Grabowski TG, Ojemann JG (2013). Local functional connectivity as a pre-surgical tool for seizure focus identification in non-lesion, focal epilepsy. *Frontiers in Neurology*.

[45] Zhong Y, Lu G, Zhang Z, Jiao Q, Li K, Liu Y (2011). Altered regional synchronization in epileptic patients with generalized tonic-clonic seizures. *Epilepsy Research*.

[46] Zeng H, Pizarro R, Nair VA, La C, Prabhakaran V (2013). Alterations in regional homogeneity of resting-state brain activity in mesial temporal lobe epilepsy.. *Epilepsia*.

[47] Mankinen K, Long X, Paakki J (2011). Alterations in regional homogeneity of baseline brain activity in pediatric temporal lobe epilepsy. *Brain research*.

[48] ILAE (1989). Proposal for revised classification of epilepsies and epileptic syndromes. Commission on Classification and Terminology of the International League Against Epilepsy. *Epilepsia*.

[49] Chao-Gan Y, Yu-Feng Z (2010). DPARSF: a MATLAB toolbox for “pipeline” data analysis of resting-state fMRI. *Frontiers in Systems Neuroscience*.

[50] Power JD, Barnes KA, Snyder AZ, Schlaggar BL, Petersen SE (2012). Spurious but systematic correlations in functional connectivity MRI networks arise from subject motion. *NeuroImage*.

[51] Song X, Dong Z, Long X (2011). REST: a Toolkit for resting-state functional magnetic resonance imaging data processing. *PLoS ONE*.

[52] Badawy RAB, Freestone DR, Lai A, Cook MJ (2012). Epilepsy: ever-changing states of cortical excitability. *Neuroscience*.

[53] David O, Guillemain I, Saillet S (2008). Identifying neural drivers with functional MRI: an electrophysiological validation. *PLoS Biology*.

[54] Schevon CA, Cappell J, Emerson R (2007). Cortical abnormalities in epilepsy revealed by local EEG synchrony. *NeuroImage*.

[55] Moeller F, Moehring J, Ick I (2013). EEG-fMRI in atypical benign partial epilepsy. *Epilepsia*.

[56] Shannon BJ, Raichle ME, Snyder AZ (2011). Premotor functional connectivity predicts impulsivity in juvenile offenders. *Proceedings of the National Academy of Sciences of the United States of America*.

[57] Fox MD, Corbetta M, Snyder AZ, Vincent JL, Raichle ME (2006). Spontaneous neuronal activity distinguishes human dorsal and ventral attention systems. *Proceedings of the National Academy of Sciences of the United States of America*.

[58] Jung KY, Kim JM, Wook Kim DW (2003). Patterns of interictal spike propagation across the central sulcus in benign rolandic epilepsy. *Clinical Electroencephalography*.

[59] Deltour L, Querné L, Vernier-Hauvette M, Berquin P (2008). Deficit of endogenous spatial orienting of attention in children with benign epilepsy with centrotemporal spikes (BECTS). *Epilepsy Research*.

[60] Rolls ET (2004). The functions of the orbitofrontal cortex. *Brain and Cognition*.

[61] Feinberg DA, Moeller S, Smith SM (2010). Multiplexed echo planar imaging for sub-second whole brain fmri and fast diffusion imaging. *PLoS ONE*.

